# Social participation and mortality: does social position in civic groups matter?

**DOI:** 10.1186/s12889-016-3082-1

**Published:** 2016-05-12

**Authors:** Yoshiki Ishikawa, Naoki Kondo, Katsunori Kondo, Toshiya Saito, Hana Hayashi, Ichiro Kawachi, for the JAGES group

**Affiliations:** Department of Health and Social Behavior, School of Public Health, The University of Tokyo, 7-3-1, Hongo, Bunkyo-ku Tokyo, 1130033 Japan; Cancer Scan, Shibuya-ku Tokyo, Japan; Center for Preventive Medical Sciences, Chiba University, Chiba, Japan; Center for Gerontology and Social Science, National Center for Geriatrics and Gerontology, Nagoya, Japan; Department of Public Health, Mccann Health Communications, Minato-ku Tokyo, Japan; Department of Social and Behavioral Sciences, Harvard School of Public Health, Boston, Massachusetts USA

**Keywords:** Japan, Social participation, Older people, Mortality, Leadership role, Propensity score

## Abstract

**Background:**

Social participation is known to predict longevity. However, little is known about the effect of social participation according to an individual’s position in civic groups. We evaluated the influence of social position on mortality, using data from a large cohort of Japanese older adults (the AGES cohort).

**Methods:**

Of 14,804 individuals aged 65 years and older enrolled in the AGES, 14,286 individuals were followed up for approximately 5 years from 2003 to 2008. We performed inverse probability of treatment weighted (IPTW) Cox proportional hazards regression with multiple imputation of missing values to compute hazard ratios (HR) for all-cause mortality according to the individual’s position in the community organization(s) to which they belonged. We examined participation in the following civic groups: neighborhood association/senior citizen club/fire-fighting team, religious group, political organization or group, industrial or trade association, volunteer group, citizen or consumer group, hobby group, and sports group or club. The values for IPTW were computed based on demographic variables, socioeconomic status, and self-reported medical condition.

**Results:**

During 22,718 person-years of follow-up for regular members of community groups and 14,014 person-years of follow-up for participants in leadership positions, 479 deaths and 214 deaths were observed, respectively. Relative to regular members, crude HR for all-cause mortality for occupying leadership positions (e.g. president, manager, or having administrative roles) was 0.72 (95 % CI:0.62–0.85). The IPTW-HR was 0.88 (95 % CI: 0.79–0.99) for participants occupying leadership positions.

**Conclusions:**

Holding leadership positions in community organization(s) may be more beneficial to health than being regular members.

## Background

As population aging has advanced in many countries worldwide, the maintenance of physical, cognitive, and social competencies of older adults has become a critical target of public health interventions. Mounting empirical evidence has suggested that the promotion of participation in community organizations is health-promoting. For example, social participation leads to lower mortality rates [1, 2], less depressive symptoms [3], and higher cognitive functioning [4] among older adults. These positive health effects may be attributable to the psychosocial benefits of social participation including enhancing self-esteem, creating purpose in life, and stress buffering [5, 6]. However, the magnitude of the impact of social participation on health varies across studies [7]; including some evidence of negative impacts on health [8–10]. As such, further understanding of the mechanism through which social participation leads to better health is warranted.

A second strand of research suggests that an individual’s status within a social hierarchy is relevant for health. According to Marmot et al, individuals occupying a higher status within an organization enjoy advantages in terms of not only material but also psychosocial resources [11]. For example, in a company setting, managers may have, in addition to higher average income, psychosocial benefits stemming from the exercise of supervisory authority as well as higher prestige. On the other hand, the possibility of reverse causality cannot be denied. For example, Boyce and Oswald reported that those with good health were more likely to be promoted [12]. In order to avoid this healthy survivor effect, some studies have used data on the prestigious award winners and nominees. For example, winning Oscar Award and Nobel Prize was associated with 4.2 years [13] and 1.6 years [14] of longevity, respectively. Since the winners and nominees can be considered as relatively homogeneous, the effect of award or higher status can be regarded as exogenous shock which enables accurate estimation of the effect of status on longevity.

However, to our knowledge, there is a dearth of empirical evidence on the effect of status within civic groups on longevity among older adults. In order to address this research gap, we use data from a large cohort of Japanese older adults which previously showed that social participation was associated with lower all-cause mortality [2]. Our hypothesis is that over and above social participation, those who participate in a civic group with leadership positions (e.g. president, manager, or having administrative roles) may enjoy greater health benefits compared to regular participants.

## Methods

### Data source

**Fig. 1:**
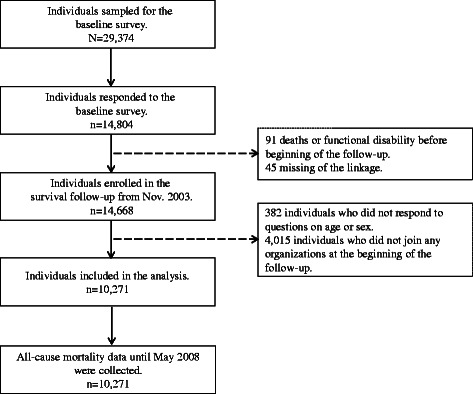
Flow chart of the study participants of the Aichi Gerontological Evaluation Study (AGES), 2003–2008

We used data from the Aichi Gerontological Evaluation study (AGES), which is a part of Japan Gerontological Evaluation Study (JAGES). The JAGES is an on-going longitudinal panel study which seeks to elucidate the social determinants of functional decline, cognitive impairment, and mortality among older adults aged 65 years and older. Figure 1 illustrates the flow chart of the study participants for this study. One wave of data were collected for this study in 2003. Of 29,374 people invited to participate, 14,804 people completed the questionnaire, corresponding to a response rate of 50.4 %. Those who did not respond with the baseline questionnaire were younger than 80 years old and were more likely to have higher household income, while there was no difference between men and women. Of the 14,804 respondents, we included 10,271 individuals in the present analysis, because 91 individuals were excluded due to deaths or functional disability before beginning of the follow-up, 45 individuals were excluded due to missing of the linkage variable before beginning of the follow-up, a further 382 individuals did not respond to questions on age or sex, and 4015 individuals did not join any civic organizations at the beginning of the follow-up. Detailed information on the design and data collection of the AGES baseline survey is available elsewhere [15, 16].

### Ethics

The JAGES protocol was reviewed and approved by the ethics committee in Research of Human Subjects at Nihon Fukushi University and has been carried out in compliance with Helsinki Declaration.

### Measurements

#### Dependent variable

All-cause mortality data until May 2008 were obtained from the six municipalities participating in the AGES cohort and were linked by the researchers using the identification number which was assigned to each study participant. One of the strength of the AGES cohort is that there is no administrative loss during approximately 5-year follow-up.

#### Independent variable

Social participation was assessed by the statement “Do you belong to the following organization or group?(yes/no)” We inquired about the following types of community organizations: neighborhood association/senior citizen club/fire-fighting team, religious group, political organization or group, industrial or trade association, volunteer group, citizen or consumer group, hobby group, and sports group or club. This questionnaire on social participation in the AGES study was derived from JGSS (Japanese General Social Survey) to make the results of the study comparable to the general population.

For those who participated in one of the above organizations or groups, the respondent’s position in the participating organization was assessed by a dichotomous (yes/no) variable with the statement: “Do you serve as the head, manager or treasurer in the organization or group?” Those who answered “yes” were categorized as “leadership position” in terms of their social positions in the participating organization, whereas those who answered “no” were categorized as “regular” members.

#### Covariates

Demographic variables included sex, age (65–69, 70–74, 75–79, 80–84, 85 years and over), marital status (married and their spouse was alive, other), and residence year (less than 5, 5–9, 10–19, 20–29, 30-39, 40-49, 50 years and more). Socioeconomic status included annual equivalized household income (less than 19,999 JPY, 20,000 JPY to 39,999 JPY”, 40,000 JPY or more”) and educational attainment (less than 6, 6–9, 10–12, 13 years and more, others). In Japan, six-three-three system of education is employed: 6 years of elementary school; 3 years of junior high school; and 3 years of high school.

In order to account for the health endowment effect, self-reported medical condition (No illness, Having illness but need no treatment, Having illness but discontinued treatment, Receiving some treatment), self-reported health status (Very good, Good, Poor, Very poor), self-reported physical condition (Very good, Good, Poor, Very poor), and Depression (Geriatric Depression Scale) [17] were considered as covariates.

### Statistical analysis

First, we evaluated the differences in mortality between the groups according to the participants’ baseline characteristics. Next, in order to address potential treatment selection bias due to the differential chances of occupying leadership position within the organizations, we used a propensity score approach in an attempt to create pseudo-populations based on the probability of assignment to treatment for estimating the causal effect. We calculated the propensity score using a logistic regression model, where the dependent variable was the position in the community organization: regular members vs. leadership position. For explanatory variables, we selected important variables that potentially affect assignment to treatment (in this instance, occupying a leadership position within an organization), namely, age, sex, annual equivalized household income, educational attainment, marital status, self-reported health, self-reported physical function, self-reported medical conditions, Geriatric Depression Scale, and residence year. After examining whether weighting balanced measured covariates between groups (Table 2), the inverse of the propensity score was incorporated to the weighted Cox proportional hazards models to calculate hazard ratios for mortality according to the social position occupied within the organization(s). This approach is an alternative to implementing propensity score matching to statistically balance for confounding variables in non-randomized studies [18, 19].

The overall proportion of missing data was 36.3 %, if we excluded participants who had missing data on at least one of the variables considered in our analysis. As such, missing data on independent variable and covariates were imputed by multiple imputation with method under the missing at random assumption. Multiple imputations based on multivariate normal model was calculated using all the covariates as explanatory variables: age, sex, annual equivalized household income, educational attainment, marital status, self-reported health, self-reported physical function, self-reported medical condition, Geriatric Depression Scale, residence year and position in the participating organization(s). We produced ten imputed data sets and the estimates were combined. SAS 9.2 (SAS Institute, Cary, NC) was used for all statistical analyses.

## Results

Fifty one percent of the participants were female. Average age of the participants was 72.7 years (Standard deviation: 5.8). Among them, 58 % of them occupied leadership roles in their participating organizations, 35 % of them were regular participants, and the positions of 7 % of them were not known due to missing response.

During 22,718 person-years of follow-up for regular members and 14,014 person-years of follow-up for those occupying leadership positions, 479 deaths and 214 deaths were observed, respectively. The mortality rate was 21.1 per 1000 person-years (95 % Confidence Interval (CI): 19.3–23.0) for regular members and 15.3 per 1000 person-years (95 % CI: 13.4–17.4) for those occupying leadership positions, respectively (Table 1).

**Table 1:** Mortality rates by baseline characteristics (*N*=10,271)

	n	(%)	Incidence/person year	Incidence rate per 1000 person-years (95 % CI)
Age				
65–69	3680	36 %	139/14,441	9.6 (8.2–11.4)
70–74	3046	30 %	160/11,855	13.5 (11.6–15.7)
75–79	2205	21 %	196/8446	23.2 (20.2–26.6)
80–84	948	9 %	143/3510	40.7 (34.7–47.8)
85 or older	392	4 %	115/1355	84.9 (71.3–101.1)
Sex				
Female	5261	51 %	255/20,498	12.4 (11.0–14.1)
Male	5010	49 %	498/19,108	26.1 (23.9–28.4)
Annual equivalized household income (JPY)				
<19,999	3304	32 %	240/12,746	18.8 (16.6–21.3)
20,000–39,999	3880	38 %	292/14,940	19.5 (17.4–21.9)
≧40,000	1023	10 %	55/3981	13.8 (10.6–18.0)
Missing	2064	20 %	166/7939	20.9 (18.0–24.3)
Educational attainment				
Less than 6 years	381	4 %	53/1418	37.4 (28.7–48.7)
6–9 years	5298	52 %	411/20,435	20.1 (18.3–22.1)
10–12 years	2964	29 %	175/11,494	15.2 (13.1–17.6)
13 years and more	1002	10 %	61/3857	15.8 (12.3–20.3)
Other	33	0 %	5/117	42.7 (18.1–100.7)
Missing	593	6 %	48/2285	21.0 (15.9–27.8)
Marital status				
Married and spouse is alive	6932	67 %	473/26,825	17.6 (16.1–19.3)
Other	2670	26 %	220/10,213	21.5 (18.9–24.5)
Missing	669	7 %	60/2568	23.4 (18.2–30.0)
Self-reported health				
Very good	861	8 %	36/3377	10.7 (7.7–14.8)
Good	6542	64 %	353/25,485	13.9 (12.5–15.4)
Poor	2126	21 %	237/8057	29.4 (25.9–33.3)
Very poor	438	4 %	101/1533	65.9 (54.6–79.6)
Missing	304	3 %	26/1154	22.5 (15.4–32.9)
Self-reported physical function				
Very good	1373	13 %	68/5372	12.7 (10.0–16.0)
Good	6844	67 %	406/26,562	15.3 (13.9–16.8)
Poor	1522	15 %	192/5721	33.6 (29.2–38.6)
Very poor	329	3 %	69/1180	58.5 (46.5–73.5)
Missing	203	2 %	18/771	23.3 (14.8–36.9)
Self-reported medical conditions				
No illness	1707	17 %	67/6709	10.0 (7.9–12.7)
Having illness but need no treatment	987	10 %	74/3817	19.4 (15.5–24.3)
Having illness but discontinued treatment	627	6 %	37/2438	15.2 (11.0–20.9)
Receiving some treatment	6323	62 %	537/24,210	22.2 (20.4–24.1)
Missing	627	6 %	38/2433	15.6 (11.4–21.4)
Geriatric Depression Scale				
Normal	6109	59 %	339/23,803	14.2 (12.8–15.8)
Mildly depressed	1944	19 %	192/7387	26.0 (22.6–29.9)
Severely depressed	348	3 %	49/1283	38.2 (29.0–50.3)
Missing	1870	18 %	173/7133	24.3 (20.9–28.1)
Residence year				
Less than 10 years	316	3 %	20/1205	16.6 (10.7–25.6)
10–19 years	439	4 %	27/1670	16.2 (11.1–23.5)
20–29 years	760	7 %	53/2926	18.1 (13.9–23.7)
30–39 years	1237	12 %	67/4814	13.9 (11.0–17.7)
40–49 years	1593	16 %	82/6207	13.2 (10.7–16.4)
50 years and more	5648	55 %	473/21,713	21.8 (19.9–23.8)
Missing	278	3 %	31/1072	28.9 (20.4–40.9)
Social participation				
Regular position in the social organization(s)	5928	58 %	479/22,718	21.1 (19.3–23.0)
Leadership position in one or more social organization(s)	3600	35 %	214/14,014	15.3 (13.4–17.4)
Missing	743	7 %	60/2874	20.9 (16.3–26.8)

Before IPTW adjustment, all of the differences in baseline characteristics comparing regular members with those occupying leadership positions were statistically significant, whereas those differences were eliminated after the inverse probability of weighting (Table 2).

**Table 2:** Relationship between social position and baseline characteristics before/after weighting the inverse probability of treatment

	Before weighting the inverse probability of treatment			After weighting the inverse probability of treatment		
	Regular position (%)	Leadership position (%)	*P* value	Regular position (%)	Leadership position (%)	*P* value
Age			<.0001			0.96
65–69	31.8	42.5		35.7	35.4	
70–74	28.3	31.9		29.6	29.4	
75–79	23.6	18.0		21.6	21.9	
80–84	11.3	5.8		9.3	9.4	
85 or older	5.0	1.8		3.8	3.9	
Sex			<.0001			0.73
Female	57.3	41.2		51.4	51.6	
Male	42.7	58.8		48.7	48.4	
Annual equivalized household income (JPY)			<.0001			0.42
<19,999	43.7	38.3		42.0	42.9	
20,000–39,999	44.6	49.3		46.1	45.3	
≧40,000	11.7	12.4		11.9	11.8	
Educational attainment			<.0001			0.86
Less than 6 years	4.8	2.1		3.8	4.0	
6–9 years	57.7	50.4		55.2	55.7	
10–12 years	28.8	33.7		30.5	30.0	
13 years and more	8.4	13.4		10.2	10.0	
Other	0.3	0.4		0.3	0.3	
Marital status			<.0001			0.35
Married and spouse is alive	67.9	78.9		71.9	71.3	
Other	32.1	21.1		28.1	28.7	
Self-reported health			<.0001			0.55
Very good	7.0	11.3		8.7	8.5	
Good	63.9	67.8		65.3	64.8	
Poor	23.8	17.8		21.6	21.9	
Very poor	5.2	3.1		4.4	4.8	
Self-reported physical function			<.0001			0.31
Very good	11.3	17.5		13.6	13.3	
Good	67.3	68.8		67.8	67.6	
Poor	17.4	11.7		15.3	15.4	
Very poor	3.9	2.1		3.3	3.7	
Self-reported medical conditions			<.0001			0.93
No illness	16.1	21.1		18.0	17.8	
Having illness but need no treatment	9.2	12.2		10.2	10.1	
Having illness but discontinued treatment	6.5	6.1		6.4	6.4	
Receiving some treatment	68.3	60.6		65.4	65.8	
Geriatric Depression Scale			<.0001			0.58
Normal	63.3	76.6		68.1	67.7	
Mildly depressed	33.6	21.9		29.3	29.9	
Severely depressed	3.1	1.5		2.6	2.5	
Residence year			<.0001			0.67
Less than 10 years	3.9	2.2		3.3	3.7	
10–19 years	5.1	3.7		4.6	4.9	
20–29 years	8.1	7.4		7.9	7.9	
30–39 years	12.9	12.8		12.9	12.9	
40–49 years	16.3	16.8		16.5	16.4	
50 years and more	53.8	57.0		54.8	54.3	

As multiple imputation was applied to the dataset, the result is based on the first of ten dataset but almost same results were acquired from the other datasets

The crude HR for mortality, relative to those who were regular members in the social organizations, was 0.72 (95 % CI: 0.62–0.85) for those occupying a leadership position in one or more community organizations (Crude model in Table 3). The IPTW-HR of mortality was attenuated to 0.88 (95 % CI: 0.79–0.99) for individuals in leadership positions (IPTW model in Table 3).

**Table 3:** Hazard ratios for all-cause mortality: results of Cox proportional hazard model (*N*=10,271)

	Crude^a)^	IPTW^b)^
	HR (95 % CI)	HR (95 % CI)
Social participation		
Regular position in the social organization(s)	reference	reference
Leadership position in one or more social organization(s)	0.72 (0.62 to 0.85)	0.88 (0.79 to 0.99)
Age		
65–69	reference	reference
70–74	1.40 (1.12 to 1.76)	1.27 (1.07 to 1.50)
75–79	2.42 (1.95 to 3.01)	2.20 (1.87 to 2.59)
80–84	4.27 (3.38 to 5.40)	3.79 (3.17 to 4.54)
85 or older	9.03 (7.05 to 11.56)	7.34 (5.84 to 9.21)
Sex		
Female	reference	reference
Male	2.10 (1.81 to 2.44)	2.75 (2.40 to 3.15)
Individual-level equivalent income (JPY)		
<19,999	reference	reference
20,000–39,999	1.01 (0.85 to 1.20)	1.19 (1.03 to 1.37)
≧40,000	0.78 (0.56 to 1.10)	1.01 (0.74 to 1.37)
Educational attainment		
Less than 6 years	reference	reference
6–9 years	0.54 (0.41 to 0.73)	0.79 (0.60 to 1.06)
10–12 years	0.41 (0.30 to 0.56)	0.78 (0.59 to 1.03)
13 years and more	0.43 (0.30 to 0.62)	0.65 (0.45 to 0.94)
Other	1.16 (0.47 to 2.91)	1.38 (0.69 to 2.75)
Marital status		
Married and spouse is alive	0.82 (0.70 to 0.97)	0.86 (0.75 to 0.99)
Other	reference	reference
Self-reported health		
Very good	reference	reference
Good	1.28 (0.91 to 1.80)	0.97 (0.72 to 1.31)
Poor	2.67 (1.88 to 3.79)	1.58 (1.13 to 2.21)
Very poor	5.98 (4.08 to 8.75)	2.55 (1.74 to 3.73)
Self-reported physical function		
Very good	reference	reference
Good	1.20 (0.93 to 1.55)	1.01 (0.79 to 1.28)
Poor	2.61 (1.98 to 3.44)	1.32 (0.98 to 1.77)
Very poor	4.59 (3.28 to 6.41)	1.48 (1.03 to 2.13)
Self-reported medical conditions		
No illness	reference	reference
Having illness but need no treatment	1.91 (1.37 to 2.66)	1.39 (1.08 to 1.80)
Having illness but discontinued treatment	1.52 (1.02 to 2.27)	1.30 (0.96 to 1.77)
Receiving some treatment	2.18 (1.70 to 2.80)	1.28 (1.04 to 1.59)
Geriatric Depression Scale		
Normal	reference	reference
Mildly depressed	1.59 (1.36 to 1.85)	1.17 (1.03 to 1.33)
Severely depressed	2.18 (1.73 to 2.74)	1.19 (0.96 to 1.48)
Residence year		
Less than 10 years	0.80 (0.51 to 1.26)	0.97 (0.69 to 1.37)
10–19 years	0.80 (0.55 to 1.17)	0.88 (0.67 to 1.16)
20–29 years	0.89 (0.68 to 1.17)	1.22 (0.97 to 1.53)
30–39 years	0.67 (0.52 to 0.86)	0.94 (0.76 to 1.15)
40–49 years	0.63 (0.50 to 0.79)	1.11 (0.93 to 1.31)
50 years and more	reference	reference

^a)^ *Crude HR* unadjusted models using each covariate as a single exposure when examining association with allcause mortality

^b)^ *IPTW HR* inverse probability of treatment weighted HR

## Discussion

The primary findings of this study of Japanese older adults was that, among those participating in community organizations, leaders who were occupying managerial or administrative positions within the organization was associated with a 12 % risk reduction of mortality independent of demographic variables, socioeconomic status, and self-reported medical condition. This was evaluated by using propensity score method.

The potential pathways linking higher social positions and health include: (1) better access to material resources to maintain health [20] and (2) psychosocial benefits attendant on occupying a higher status position [11, 21]. While higher position within certain social structures (such as a workplace) can determine access to material resources such as higher incomes, stock options, fringe benefits (e.g. housing allowances), and more generous pension benefits, these are unlikely to apply to older people occupying higher positions within civic (mostly voluntary) groups. Instead, occupying a leadership position within a community organization is likely to be associated with psychosocial benefits, such as prestige, meaningful social role, sense of purpose, psychological well-being and decision-making authority. For example, past research using AGES cohort data supports this idea that older women with roles involving responsibility in community organizations enjoyed better mental health compared to those participating as regular members [22].

Another potential mechanism through which higher social position leads to lower all-cause mortality is the theory of communication inequality [23, 24]. According to the theory [23], communication inequalities are defined as “differences among social classes in the generation, manipulation, and distribution of information at the group level and differences in access to and ability to take advantage of information at the individual level [p. 287].” For example, those in leadership position might be more likely to be exposed to health information than those in regular position, which lead them to make healthy decision makings decisions. However, little empirical studies have examined whether such inequality in exposure to health information exists in the context of social participation among older adults. Further study on the characteristics of those in leadership position compared with those in regular position may contribute to the understanding of the effect of status on health.

This study has several limitations. First and foremost, we cannot completely eliminate the possibility that there is selection of individuals into both social participation and leadership roles (i.e. healthy and robust individuals join more groups, and they are more likely to volunteer for leadership roles in the groups which they belong to). Although we carefully controlled for selection using the IPTW approach, we were only able to take account of observed variables in the dataset. It is still possible that respondents differed with respect to unobservable factors influencing both their participation and their subsequent mortality risks (i.e. residual confounding). For example, past research using panel data shows that the person’s long-term unemployment history is negatively associated with health status [25], which might reduce the chance of occupying leadership position. Future AGES cohort study should incorporate these known potential factors in their new wave of data collection.

Secondly, our measures of social participation remained crude. For example, Aida et al have reported that horizontal (as compared to vertical) organizations had beneficial effects on oral health in older Japanese adults, using AGES data [26]. We hypothesized that the same phenomenon can be applied to the result of this study that the effect of social position on mortality differs by type of the organizations. In the AGES study, the question on social position was as follows: “Do you serve as the head, manager or treasurer in the organization or group?” Therefore, we could not know which type of organization the respondents serve in a leadership capacity. Also, our measure of social participation does not capture the intensity of involvement. Further study is warranted to uncover the interactive effect of the social position and the type of/the intensity of involvement in the community organization on health.

Third, we could not measure the change in status within the participating organization or the change in organizational membership during the follow-up period, since the measurement was carried out just once at the beginning of the study. Future study should use multi-wave panel data to take this aspect into account.

Fourth, long-term effects of participation in community organizations beyond the period of 5-year follow up could not be evaluated. Past longitudinal study reports that the impact of increasing social status on psychological wellbeing persists over 5 years [27]. As such, it is an interesting research question to examine how long the effect of increasing social status will last. Future study with longer follow-up period should examine whether similar results can be obtained or not.

Fifth, the existence of non-respondents (response rate: 50.4 %) could be a source of potential selection bias, resulting in either over- or under-estimation of the effect of social position on all-cause mortality.

In summary, our results emphasize the importance of simultaneously considering the social position of individuals within the organization when examining the influence of social participation on health. Over and above social participation, findings of our study suggest that promoting older Japanese people to leadership position as many as possible may have positive impact on longevity. Nonetheless, it is plausible that taking leadership in a group may provide health benefits only for selected persons with specific characteristics, e.g., personality and socioeconomic statuses. Further study should investigate the interaction between group leadership and those factors on health.

## Conclusions

Holding leadership positions in community organization(s) may be more beneficial to health than being regular members.

### Ethics

Our study protocol and informed consent procedure were approved by the Ethics Committee on the Research of Human Subjects at Nihon Fukushi University.

### Availability of supporting data

The dataset supporting the conclusions of this article is available in response to the request from the researchers admitted by the JAGES committee.
